# Components of the human-specific, p53-mediated “kill switch” tumor suppression mechanism are usurped by human tumors, creating the possibility of therapeutic exploitation

**DOI:** 10.20517/cdr.2019.89

**Published:** 2019-12-19

**Authors:** Jonathan Nyce

**Affiliations:** Clinical Research, ACGT Biotechnology, Collegeville, PA 19426, USA.

**Keywords:** p53, TP53, dehydroepiandrosterone, dehydroepiandrosterone sulfate

## Abstract

We recently reported our detection of an anthropoid primate-specific, adrenal androgen-dependent, “kill switch” tumor suppression mechanism that is triggered by the inactivation of the p53 tumor suppressor. This mechanism reached its highest expression only in humans as a result of the human-specific harnessing of fire, which resulted in an extraordinary increase in exposure to polycyclic aromatic hydrocarbons. This “kill switch” becomes inoperative in modern humans once they exceed the primitive human lifespan of 25-30 years, because lifespan has more than tripled in modern times, but the secretion curve for dehydroepiandrosterone sulfate remains fixed at the level required for the primitive human lifespan. Components of this “kill switch” are consequently usurped by human tumors, and these are already targets for inhibition in cancer chemotherapy. Here, we suggest a different strategy: using the usurped components of the kill switch to activate prodrugs, rather than as targets for inhibition. This strategy is in its infancy, but has the potential to enable more tumor-specific cytotoxicity, which the inhibition strategy generally cannot achieve. Detection of the usurpation of kill switch elements in liquid biopsy analyses enables the collection of information relevant to this new class of tumor biomarkers without the necessity of invasive tissue biopsy.

## Introduction

**Figure 1:**
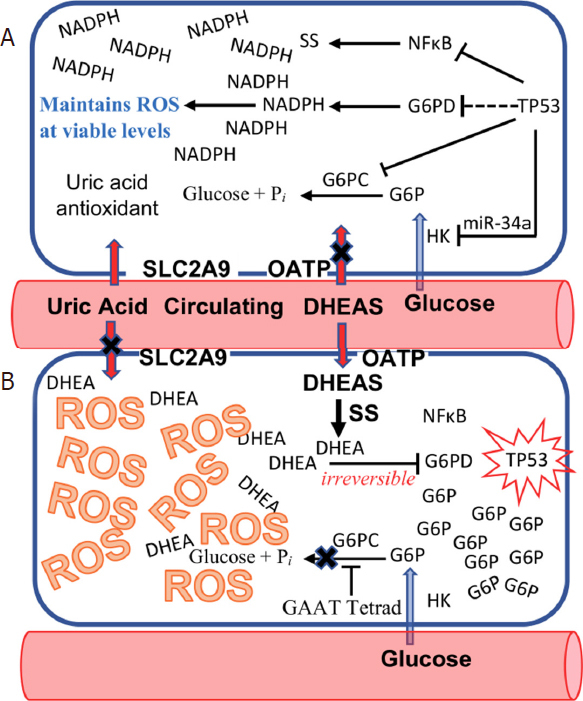
The species-specific “Kill Switch” tumor suppression mechanism of humans. A: normal cell with active TP53; B: inactivation of TP53 triggers importation of dehydroepiandrosterone sulfate (DHEAS) into the affected cell, which is de-sulfated to dehydroepiandrosterone (DHEA), an uncompetitive inhibitor of glucose-6-phosphate dehydrogenase (G6PD). Simultaneously, glucose-6-phosphate (G6P) accumulates in the affected cell, due to an anthropoid primate-specific sequence motif (GAAT tetrad) in the G6PC promoter and importation of uric acid antioxidant into the cell by SLC2A9 ceases. The accumulation of both DHEA and G6P in the cell drive the uncompetitive inhibition of G6PD to become irreversible, leading to reactive oxygen species (ROS)-mediated cell death, extinguishing TP53 inactive cells at the single cell stage, before they can evolve into the heterogeneous tumor cell populations that have made cancer incurable up to now

Dehydroepiandrosterone sulfate (DHEAS) has been a critical feature of primate evolution since the inception of this lineage. We have recently reported an anthropoid primate-specific, DHEAS-dependent, p53-mediated “kill switch” tumor suppression system that reached its culmination in humans, which have by far the highest peak levels of DHEAS, evolving as a countermeasure to offset increased carcinogen exposure resulting from the harnessing of fire. This “kill switch” is triggered by the inactivation of the p53 tumor suppressor, which causes circulating DHEAS to be rapidly imported into the affected cell, where it is de-sulfated to DHEA by steroid sulfatase (STS)^[1]^ **[Figure 1]**. DHEAS is unique in intermediary metabolism in that its proximate metabolite, DHEA, is an uncompetitive inhibitor of glucose-6-phosphate dehydrogenase (G6PD), a critical enzyme among whose functions is the generation of the intracellular NADPH required to maintain reactive oxygen species (ROS) to levels survivable by the cell. Uncompetitive inhibition is otherwise unknown in intermediary metabolism because, in the presence of high intracellular concentrations of substrate and inhibitor, it rapidly becomes irreversible^[2]^. While high intracellular levels of DHEA can be generated by the import of DHEAS from the circulation, the accumulation of high intracellular levels of glucose-6-phosphate (G6P) substrate are impossible in non-anthropoid species because the synthesis of vitamin C *via* gulonolactone oxidase (GLO), and the activity of glucose-6-phosphatase (G6PC), both act as “sinks” for G6P, preventing such accumulation. Anthropoid primates, including humans, show an Alu transposon-mediated inactivation of GLO and a specific promoter modification in the G6PC promoter that together enable G6P to accumulate in p53-affected cells, sufficient to drive the uncompetitive inhibition of G6PD by DHEA to irreversibility^[3]^. Irreversible uncompetitive inhibition of G6PD by DHEA in cells with inactivated p53 thus leads to a catastrophic increase in ROS, extinguishing such cells at the single cell stage before they have the opportunity to grow into the heterogeneous tumor cell populations that have made cancer incurable up to now. If the DHEAS-dependent, p53-mediated “kill switch” represents an effective tumor suppression mechanism, why is lifetime cancer risk in our species an astonishing 40%, tenfold higher than in other large, long-lived animals? The answer to this question is that evolution has not had sufficient time to keep pace with increases in the modern lifespan. Thus, throughout 99.95% of our species existence, the human lifespan was 25-30 years. In a display of physiological economy, circulating levels of DHEAS therefore peak at 25 years of age, and rapidly decline thereafter. The DHEAS-dependent, p53-mediated kill switch tumor suppression mechanism evolved to protect our species during this lifespan. Modern humans live more than three times longer, and therefore do so without the protection of the species-specific tumor suppression system, since circulating levels of DHEAS fall to levels incapable of supporting kill switch function when, by modern standards, we are at the inception of our adult lives.

Because the kill switch is nonfunctional in aging humans, tumor cells are free to usurp certain of its components for their own use. A review of tumor gene expression registries shows many kill switch components that have been appropriated by tumors as elements of their growth trajectories. Many of these kill switch components have been identified as targets for inhibition in cancer chemotherapy strategies. We propose an opposite strategy, using kill switch components appropriated by tumors to activate novel prodrugs to their cytotoxic forms. We discuss elements of this strategy and show how it might enable tumor-specific cytotoxicity that is not possible employing inhibition strategies.

## Examples of “kill switch” components appropriated by human tumors: DHEAS transport proteins and steroid sulfatase

In contrast to DHEA, which can cross cellular membranes, DHEAS requires transport across cell membranes by specific transport proteins. In addition, unlike DHEA, DHEAS is not an effective inhibitor of G6PD until it is de-sulfated to DHEA. Thus, Circulating DHEA must be maintained at very low serum concentrations, orders of magnitude below its inhibition constant for G6PD (*K*_*i*_ = 18.5 μmol/L; compare DHEAS *K*_*i*_ = 310 μmol/L^[4]^; peak serum concentrations of DHEA ≈ 30 nmol/L, and of DHEAS ≈ 11.5 µmol/L^[5]^). While DHEAS may have fallen below levels required for triggering of the kill switch tumor suppression system in aging individuals, circulating DHEAS, which serves as a precursor for dihydrotestosterone and estrogen synthesis, remains problematic for hormone-dependent cancers^[6]^. Locally advanced or metastatic prostate cancer, for example, is treated with androgen deprivation therapy (ADT), which can be counteracted by circulating DHEAS^[7]^. While patients generally respond well to ADT, such tumors invariably progress to castration-resistant prostate cancer^[8]^. Such failure is generally attributed to intra-tumoral androgen synthesis, and DHEAS has been demonstrated to be the major precursor for androgen synthesis, particularly dihydrotestosterone^[9,10]^. Circulating DHEAS is imported into tumor cells by *SLCO*-encoded organic anion transporting polypeptide (OATP) transporters, and the expression of certain of these transporters has been demonstrated to be induced under androgen-depleted conditions^[11,12]^. Accordingly, inhibitors of OATP transporters are considered high priority targets in anti-cancer drug discovery programs in hormone dependent neoplasias^[13,14]^.

Various human cancers - both those typically known to be endocrine-dependent, as well as non-endocrine cancers - show high expression of STS; i.e., they have appropriated this element of the kill switch for their own purposes, able to do so because circulating levels of DHEAS have fallen below those required for kill switch triggering^[15,16]^. There is not a straightforward solution to the problem of DHEAS levels that have declined to levels that cannot support kill switch triggering - such as elevating them to peak levels - because clinical tumors have evolved substantial heterogeneity by the time they are detected, which can obfuscate or override kill switch function. Hormone-dependent cancers might therefore be stimulated to grow and metastasize by reconstitution of peak levels of DHEAS, without triggering the natural kill switch because its mechanism has been overridden by genetic and epigenetic changes that have occurred within the tumor. Also, G6PD is an oncoprotein, for example, capable of directly inhibiting p53. When non-tumorigenic mouse cells are supplemented with large amounts of human G6PD, such cells become transformed and capable of producing tumors. G6PD is over-expressed in the majority of human tumors, and such over-expression presents an additional hurdle. Nevertheless, as discussed below, in tumors that have appropriated certain elements of the kill switch, potentially effective strategies for the induction of tumor-specific toxicity present themselves.

**Figure 2:**
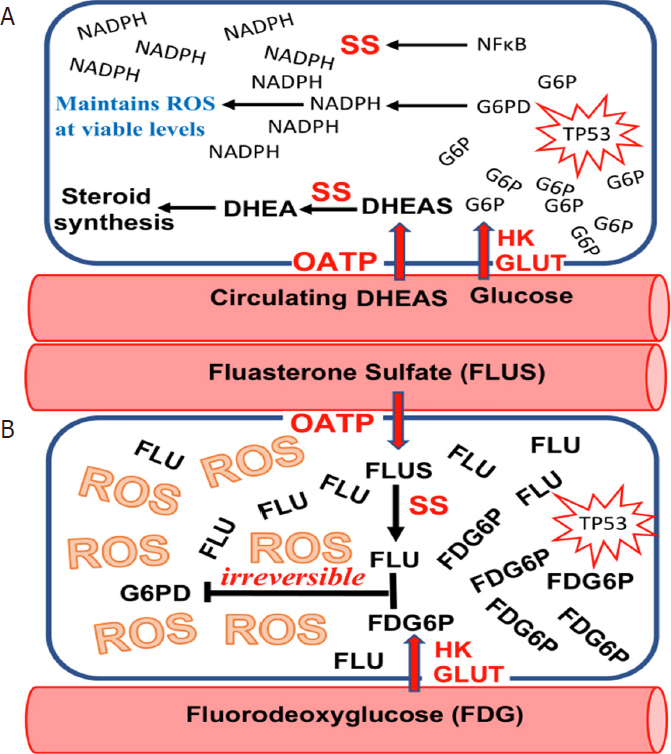
When the “Kill Switch” tumor suppression mechanism fails due to age-related decline in circulating dehydroepiandrosterone sulfate (DHEAS), many human tumors are subsequently able to appropriate elements of the “Kill Switch” for their own use. A: organic anion transporting polypeptide (OATP) that transport DHEAS into the cell, and SS that de-sulfates DHEAS to dehydroepiandrosterone (DHEA) are two examples of “Kill Switch” elements that are frequently usurped by human tumors; B: such appropriation of “Kill Switch” elements by human tumors makes them susceptible to selective killing by such drugs as fluasterone sulfate, a fluorinated analog of DHEAS that cannot be metabolized to steroid hormones, and is therefore of particular use in SS-expressing hormone sensitive tumors. Most human tumors may be rendered sensitive to fluasterone or fluasterone sulfate treatment by pretreatment with fluorodeoxyglucose (FDG), a fluorinated analog of FDG that is selectively taken up by human tumors, where it accumulates. FDG is metabolized to FDG6P, a fluorinated analog of glucose-6-phosphate (G6P)

Similar to OATPs, STS has become an active target for inhibitor synthesis, in an attempt to prevent circulating DHEAS from contributing to tumor growth^[17,18]^. As with OATPs, we propose an alternative strategy: using STS appropriated by various tumors to activate novel compounds to their tumoricidal forms^[19]^ **[Figure 2]**.

## Drug discovery and development based upon tumor-appropriated kill switch elements

### Fluasterone sulfate (3β-dehydroxy-16α-fluoro-DHEA sulfate) [Figure 3]

Fluasterone (3β-dehydroxy-16α-fluoro-DHEA) was studied both by the National Cancer Institute for potential tumor preventative effects^[20]^ and by Aeson therapeutics for a variety of other human conditions such as traumatic brain injury, cardiovascular disease, diabetes, and obesity^[21]^. It was eventually abandoned for clinical evaluation due to a narrow therapeutic index. Fluasterone has the important feature that, unlike DHEA, it cannot be used as a precursor for steroid hormone synthesis^[22,23]^. It is also approximately 30-fold more potent than DHEA as an uncompetitive inhibitor of G6PD^[24,25]^, but this increase in bioactivity was offset in clinical testing by the corresponding increase in toxicity to nontarget tissues. We point out that these studies on fluasterone were performed before our discovery of the kill switch tumor suppressor mechanism, which casts prior preclinical and clinical results in a completely different light. Being a more potent inhibitor of G6PD than DHEA presented obstacles to the therapeutic use of fluasterone. To overcome this liability, we synthesized the sulfate form, producing 3β-dehydroxy-16α-fluoro-DHEA sulfate (fluasterone sulfate). Unlike fluasterone, fluasterone sulfate is not toxic, showing a toxicity profile similar to DHEAS. However, when imported into cells actively expressing STS, fluasterone sulfate is rapidly converted to fluasterone, which is highly toxic. We have proposed a series of clinical studies to the U.S. National Cancer Institute to deploy fluasterone sulfate first in canine tumors that highly express one or more of the DHEAS transport proteins and STS. Inflammatory mammary carcinomas are the most aggressive mammary cancers in both women and dogs, and both are known to have high intratumor levels of DHEA^[26]^. Our rationale for these studies is that fluasterone sulfate will be selectively taken up by such canine tumors, and then selectively metabolized to highly toxic fluasterone, triggering the kill switch in a much more selective fashion than was possible in the initial NCI studies on fluasterone. A successful study in canine spontaneous inflammatory mammary carcinoma would encourage moving toward a similar study in women with this disease, as well in DHEAS-sensitive prostate carcinoma expressing high levels of DHEAS transport proteins and STS.

**Figure 3:**
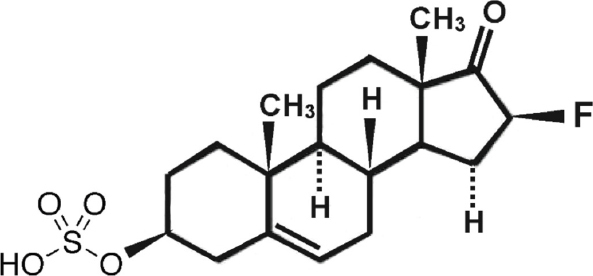
Structure of fluasterone sulfate

## Fluorodeoxyglucose [Figure 4]

An additional “kill switch” strategy is to take advantage of the avidity for glucose shown by most tumors. This avidity has already been exploited for the purpose of visualizing tumors, using the glucose analog 18F-fluorodeoxyglucose (18F-FDG)^[27]^. Similar to glucose, FDG is taken up by an array of human tumors, whereupon it is metabolized to 18F-FDG-6-Phosphate, an analog of G6P. FDG-6-P supports uncompetitive inhibition of G6PD by DHEA (or fluasterone), but binds so strongly to G6PD that very little 6-phosphogluconate product results. Accordingly, when 18F-FDG is administered to patients, it preferentially accumulates in tumor tissues, often dramatically so, enabling the identification and semi-quantitative analysis of primary neoplasms and metastases using F18 positron emission tomography^[28]^. In a series of studies proposed to the NCI, 18F-FDG will be used to qualitatively and quantitatively evaluate primary and metastatic tumor load in spontaneous canine tumors, with those tumors showing extreme 18F-FDG uptake becoming candidates for treatment with fluasterone (fluasterone sulfate if they also show high expression of STS). Tumors showing high avidity for 18F-FDG will subsequently be treated with unlabeled high dose FDG, followed by fluasterone or fluasterone sulfate, depending upon STS expression. Our strategy is that, by accumulating FDG-bound G6PD preferentially in tumor cells, uncompetitive inhibition of G6PD by fluasterone will be driven to reach irreversibility preferentially in tumor cells. In this way, the therapeutic index of fluasterone can be improved. In tumors that both avidly take up FDG and express high levels of STS, therapeutic index can be further optimized. Here, too, we are proceeding with studies in dogs with spontaneous tumors as a model system for human cancer.

**Figure 4:**
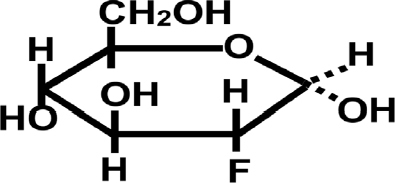
Structure of fluorodeoxyglucose

## Discussion

The existence of species-specific mechanisms of tumor suppression requires reappraisal of common laboratory animals in the construction of model systems with which to study human cancer. We argue that, because of the existence of such species-specific mechanisms of tumor suppression, one vertebrate species cannot be used to construct a valid model system of cancer in another vertebrate species^[1,3]^. However, although imperfect, dogs with spontaneous cancer offer perhaps the best non-human animal model available, because they possess a rudimentary form of an otherwise anthropoid primate-specific “kill switch” tumor suppression system based upon circulating DHEAS (which may have enabled their co-habitation with humans, and resistance to co-exposure to polycyclic aromatic hydrocarbons resulting from human harnessing of fire)^[1,3]^. We have demonstrated that failed “kill switches” can still be triggered to fire in some canine tumors, and have proposed a collaboration with NCI to expand those studies in canine cancer to include the strategies discussed above, employing fluasterone sulfate for STS-expressing canine tumors, and with FDG to exploit the “kill switch” kinetics of irreversible uncompetitive inhibition of G6PD in canine tumors that avidly take up FDG. A recent clinical study of DHEA (100 mg/day) in advanced metastatic breast cancer showed virtually no activity. This is what we would predict, based upon the lack of specificity of administered DHEA for tumor tissue^[29]^. To the extent that some of the tumors in those patients expressed significant levels of OATP transport proteins and STS, fluasterone sulfate could be predicted to have shown a much greater degree of tumor-specific toxicity. To the extent that some of those same tumors showed enhanced uptake of FDG, selective induction of toxicity in tumors might be expected to be still further increased.

Liquid biopsy represents a relatively non-invasive method to assess the presence of tumor biomarkers^[30]^. In recent work, successful liquid biopsy of mRNA in single circulating tumor cells^[31,32]^, and even in circulating tumor mRNA has been used to successfully predict treatment outcome^[33]^. Shen *et al*.^[34]^, for example, recently reported that plasma levels of BRCA1 mRNA predicts sensitivity of advanced gastric cancer to platinum, docetaxel, and pemetrexed, while plasma levels of TOPOI mRNA predicted sensitivity to irinotecan. The newly identified adrenal androgen-mediated, p53-dependent, human-specific “kill switch” tumor suppression mechanism^[35]^, offers additional targets for liquid biopsy. We predict that components of the “kill switch” tumor suppression system that have been appropriated by human tumors represent biomarkers that can be used to guide treatment strategies using new classes of drugs that act via the “kill switch” mechanism.

## Declarations

### Copyright

© The Author(s) 2019.
